# Creatinine standardization: a key consideration in evaluating whole blood creatinine monitoring systems for CKD screening

**DOI:** 10.1007/s00216-022-03942-7

**Published:** 2022-03-09

**Authors:** Raymond Neil Dalton, Timothy Scott Isbell, Ryan Ferguson, Louis Fiore, Andrei Malic, Jeffrey Anton DuBois

**Affiliations:** 1 WellChild Laboratory, Evelina London Children’s Hospital, London, UK; 2 Department of Pathology, Division of Laboratory Medicine, Saint Louis University School of Medicine, St. Louis, MO USA; 3 Department of Medicine, Section of General Internal Medicine, Boston University School of Medicine, Boston, MA USA; 4 Department of Epidemiology, Boston University School of Public Health, Boston, MA USA; 5 Adirondack Clinical Research Associates, Cambridge, UK; 6 Medical and Scientific Affairs, Nova Biomedical, Waltham, MA USA; 7 Adirondack Clinical Research Associates, Poland, NY USA

**Keywords:** Stable isotope dilution liquid chromatography mass spectrometry, Creatinine, Traceability, Standardization

## Abstract

Early detection of CKD using point of care creatinine and eGFR testing improves patient management outcomes. We undertook a field study to evaluate the use of a whole blood creatinine/eGFR device to screen a rural Nicaraguan population to determine the variability between creatinine methods and specimen types. All specimens including capillary and venous dried blood spots (DBS) were tested with an isotope dilution liquid chromatography mass spectrometry (ID-LCMS) gold standard method. This is to our knowledge the first time a capillary whole blood (POC) method has been directly compared to the gold standard IDMS method, through the novel approach of using dried blood spots. Capillary and venous whole blood specimens were obtained and tested directly with the BCMS method, and then, DBS samples were prepared. Venous plasma specimens were tested using three laboratory analyzer creatinine methods. DBS were sent to the site performing ID-LCMS. Control samples were also prepared to assess the stability of shipment and storage of DBS. The ID-LCMS method was aligned using primary and secondary standards. Sixty-six (66) patients participated in the study, and the CKD prevalence rate was 7.8%. While all creatinine methods showed a good correlation to ID-LCMS, there was a positive bias (mean absolute bias range: 0.21–0.63 mg/dL). All methods used were 100% sensitive, but specificity varied from 62.7 to 94.9% with PPV ranging from 25 to 62.5%. A correction factor was used to align the values from each method to ID-LCMS which improved the specificity of each method. This study used a unique DBS approach to align capillary whole blood creatinine to ID-LCMS. To ensure reliability of BCMS for identifying screened patients with CKD, it is important to establish IDMS traceability and alignment prior to undertaking CKD studies.

## Introduction

Chronic kidney disease (CKD) is now recognized as a major global public health problem [1, 2]. CKD can have a significant clinical impact at the individual patient level, resulting in decreasing quality of life and reduced life expectancy, and also at the population level, by increasing healthcare costs and the demand for healthcare services [3]. In the United States (US), simulation modeling indicates that the prevalence of CKD in adults 30 years or older is projected to increase considerably over the next 15 years [4]. In Europe, the prevalence of diabetic stage 5 CKD is predicted to increase annually by 3.2% in the next 10 years [5]. The increase in prevalence of type 2 diabetes mellitus (T2DM) has also impacted the incidence of CKD, particularly in the US, with estimates suggesting close to 40% of patients with T2DM developing CKD [6, 7]. This has led to an increasing incidence of end-stage renal disease (ESRD) as the prevalence of T2DM increases [8]. Globally, the number of patients requiring renal replacement therapy (RRT) will exceed five million by 2030 [9], adversely impacting public and private healthcare costs [8, 10].

Based on the significant impact of CKD, the Kidney Disease Outcomes Quality Initiative (KDOQI) and Kidney Disease: Improving Global Outcomes (KDIGO) recommend focusing on early identification of CKD [11]. Despite guideline recommendations, testing for CKD in adults with diabetes and hypertension is low in routine clinical care [12] and even lower in rural populations [13]. Identifying CKD at an early stage will initiate early patient management and treatment to slow disease progression and reduce the long-term morbidity of CKD, which could reduce the high healthcare costs associated with RRT. Screening for CKD may be particularly cost-effective in patients with diabetes and hypertension, and in populations with higher risk of CKD [14]. The key to early identification of at-risk who are often-asymptomatic patients is the implementation of screening programs in the community and primary care settings. This includes a role for simple-to-use, point-of-care (POC) creatinine methods, which potentially can be used for creatinine self-monitoring similar to self-monitoring blood glucose or blood ketone devices. The use of a POC whole blood creatinine monitoring system (BCMS) has been shown to be feasible and cost-effective in primary care settings and pharmacies, providing improvements to clinical services and cost savings in patient care [15, 16, 17]. However, a crucial issue in both evaluating and using a BCMS in CKD prevalence studies is the level of standardization of the test and reference methodology. A systematic review of CKD prevalence studies showed considerable variation in the creatinine methods used and especially in the method of calibration alignment, with very few of the methods reported as aligned to a stable isotope dilution mass spectrometry (IDMS) true reference method [18].

Differences in creatinine assays can impact the outcome of CKD prevalence studies; for example, Jaffe methods can overestimate serum creatinine and therefore overestimate CKD prevalence [19]. Although recommendations for IDMS standardization have been promoted for several years to reduce the systematic bias in creatinine determination and to increase inter-laboratory comparability [20], many different Jaffe methods continue to be used routinely for laboratory testing without IDMS alignment for epidemiological studies, resulting in confusing study findings [21].

CKD represents a major challenge in Latin American countries because of the high incidence, clinical burden to the population with increasing prevalence of RRT, and consequent impact on healthcare costs [22]. Variable prevalence rates of CKD have been reported in Nicaragua, with high rates seen in male agricultural workers, miners, and older individuals [23, 24, 25]. Screening for CKD in countries like Nicaragua could play an important role in reducing long-term healthcare costs that could overburden healthcare infrastructure [22]. An assessment of the reliability of a BCMS (StatSensor Creatinine Hospital Meter System, Nova Biomedical, Waltham, MA, USA) for creatinine measurement in a Nicaraguan patient population has previously been reported [26]. This BCMS has been reported to be accurate for detecting pathological creatinine values in patients [27, 28], and reliable for monitoring kidney function after renal transplantation [29, 30]. The Nicaraguan study reported that the BCMS demonstrated acceptable repeatability and excellent sensitivity (100%) but reported modest specificity (79%) for identifying subjects with abnormal creatinine [26]. The laboratory method used was a modified Jaffe that was not IDMS aligned for the purposes of this study. The study design did not involve IDMS alignment of any of the methods including the BCMS so we could better understand the bias differential between methods. However, during the study, additional capillary and venous samples were collected and tested by additional laboratory methods including an ID-LCMS method.

The aim of this extensive study was to evaluate the inherent variability of the study creatinine methods and specimen types, which can confound prevalence studies in the field. Secondarily, our objective was to determine the degree of variability between plasma and whole blood methods relative to the IDMS method. Myers et al. [20] suggest that the use of NIST SRM 957 standards should reduce or eliminate method variability but use of this standard set cannot be applied to whole blood creatinine methods [31, 32]. This is to our knowledge the first time a capillary whole blood (POC) method has been directly compared to the gold standard IDMS method, through the novel approach of using dried blood spots.

## Materials and methods

### Patient enrollment and specimen collection

Whole blood specimens were obtained by venipuncture and collected in tubes containing lithium heparin anti-coagulant from patients attending outpatient clinics at the hospitals in the municipalities of Rivas and San Juan del Sur, Nicaragua. The Institutional Review Board of the Dirección General de Docencia e Investigación of the Ministry of Health of Nicaragua approved the study. Each venous whole blood specimen was directly tested in duplicate using the BCMS according to the manufacturer’s instructions (StatSensor Creatinine Hospital Meter System, Nova Biomedical, Waltham, MA, USA). Following testing on the day of specimen collection, a volume of each venous whole blood specimen was added to a properly identified and labeled Whatman 903 Sample Collection Card (GE Healthcare) sufficient to fill a circle spot on the card, labeled as venous, allowed to dry as a DBS, and protected from contamination using the wrap around cover. The heparinized tubes were sent refrigerated to the national reference laboratory in Managua (Centro Nacional de Diagnóstico y Referencia) for testing. Following venous whole blood creatinine testing (in duplicate) on the BCMS, plasma was separated by centrifugation and tested in duplicate using the Cobas Integra 400 analyzer’s (Roche Diagnostics, Mannheim, Germany) modified creatinine Jaffe non-enzymatic method. All testing was performed within 24 h of blood collection. Plasma samples were stored at − 20 °C, shipped frozen to Nova Biomedical within 3 days. The sample were tested 2 weeks later after samples were thawed, thoroughly mixed prior, and aliquots were prepared for testing on the Dimension RxL (Siemens Healthcare, Erlangen, Germany) and the Vitros DT60 (Ortho Clinical Diagnostics, Rochester, NY, USA) enzymatic methods. As previously reported, creatinine is stable when frozen and retested after the sample was thawed and properly mixed [33].

Following venipuncture, capillary whole blood (3–6 μL) was obtained from each patient using a small lancet to puncture the fingertip (Dynarex Sensilance 21-Gauge Safety Lancets, Orangeburg, NY, USA). A drop of capillary blood was applied to the BCMS per the manufacturer’s instructions. The BCMS measurements were performed in duplicate using capillary whole blood from the same fingerstick. Following BCMS testing, capillary whole blood from each patient was applied to the Whatman 903 Sample Collection Card sufficient to fill a circle spot, labeled as capillary, allowed to dry at room temperature, and protected from contamination. The venous and capillary whole blood DBS were then sealed in a plastic bag, stored at 2–8 °C, and shipped at ambient temperature to the WellChild Laboratory at Evelina London Children’s Hospital in London, UK. On receipt, the DBS samples were stored at − 80 °C until analysis by ID-LCMS. Drying conditions were replicated in the IDMS laboratory to validate the stability of room temperature drying conditions.

### Creatinine measurement methods

Liquid chromatography stable isotope dilution electrospray mass spectrometry was performed on an AB SCIEX API 5000 (Applied Biosystems, Warrington, UK), as previously described [34, 35]. Venous specimens were centrifuged to obtain plasma for testing on the three laboratory analyzer creatinine methods: (1) Cobas Integra 400 analyzer utilizing a compensated Jaffé Generation 2 non-enzymatic method, (2) Dimension RxL utilizing an enzymatic method, and (3) Vitros DT60 utilizing an enzymatic method. Capillary and venous whole blood specimens were tested in duplicate on the BCMS, which utilizes an enzymatic amperometric method employing an enzyme cascade reaction. The study design involved comparison of whole blood creatinine measurements to plasma creatinine measurements using three laboratory methods, the BCMS, and LC IDMS DBS.

### ID-LCMS assay standardization and control

For LCMS assay standardization, three levels of aqueous creatinine standards were prepared from the National Institute of Standards and Technology (NIST) primary solid standard 914a. For LCMS assay quality control (QC), certified reference materials, NIST serum reference material (SRM) 957a I and II, RELA external QC materials, RELA 2009 A and B, and three levels of “in-house” prepared plasma controls were prepared and assessed. In addition, control DBS were prepared from fresh heparinized whole blood samples obtained from two normal volunteers: whole blood was applied to Whatman 903 Sample Collection cards, sufficient to fill 20 circles. The control samples were allowed to dry, protected from contamination, then sealed in a plastic bag, stored at 2–8 °C for 24 h, restored to room temperature (RT) for a further 48 h, and then stored at − 80 °C until analysis. This sequence was replicated for patient DBS sample shipment and storage.

### ID-LCMS analysis of dried blood spots

The venous, capillary, and control DBS cards were removed from the freezer and allowed to come to RT (22 °C) before manual punching of 6 mm of DBS (nominal 10 µL) into 2-mL round-bottomed polyethylene Eppendorf tubes. The tubes were capped and the samples left at RT overnight before extraction and analysis the following day. To each DBS, 10 µL of deionized water was added, followed by 50 µL of 25 µmol/L ^2^H_3_-creatinine. After an initial 10-s vortex mix, the samples were mixed gently at RT for 20 min before the addition of 200 µL of acetonitrile. Samples were then vortex mixed for 10 s and centrifuged at 21,000 rpm for 4 min. Supernatants, 200 µL each, were transferred to a 96-deep-well polypropylene block, covered with a sealing mat, and transferred to the auto-sampler for ID-LCMS analysis. All the prepared patient samples were tested in a single analysis run.

The preparation of the control DBS was used to compare whole blood creatinine, plasma creatinine, and DBS creatinine in the two subjects. In a single assay, for each subject, 10 plasma, 10 whole blood, and 10 DBS were analyzed in the sequence subject 1 plasma 1, subject 1 whole blood 1, subject 1 DBS 1, subject 1 plasma 2, ……….subject 1 plasma 10, subject 1 whole blood 10, subject 1 DBS 10, subject 2 plasma 1, ………subject 2 DBS 10.

### Data analysis

Data were analyzed using Analyse-It and Windows Excel® statistical method packages. Linear regression analysis was used for determination of correlation coefficient, intercept, and slope. Differences in water volume between plasma and whole blood measurements of creatinine were corrected using simple linear regression as previously described by the NKDEP Working Group on Commutability of Whole Blood Methods^32^*.* The mean bias, standard deviation (SD), and percent bias difference between the results of the laboratory/POC testing methods and the results of the ID-LCMS method were calculated. An ID-LCMS creatinine result greater than the upper limit of a normal reference interval (0.5–1.2 mg/dL) was considered abnormal and was used as a decision point for assessing the sensitivity and specificity of the methods to identifying individuals at risk for CKD. Sensitivity, specificity, positive predictive value (PPV), and negative predictive values (NPV) were calculated for the ability of each method to detect creatinine, as defined by an IDMS creatine cutoff value of 1.2 mg/dL A correction factor baserd on the mean bias difference between each laboratory and POC method and the IDMS gold standard method was used to realign results to the gold standard IDMS method.

## Results

### Study population

A total of 66 patients participated in the study ranging in age from 24 to 88 years, of which 42 were adult females, 20 were adult males, and four patients’ genders were not recorded. Venous DBS ID-LCMS creatinine values ranged from 0.4 to 8.9 mg/dL (mean 0.85 mg/dL, sd 1.10 mg/dL) and capillary DBS ID-LCMS creatinine values ranged from 0.4 to 9.4 mg/dL (mean 0.83 mg/dL, sd 1.19 mg/dL). Five patients with creatinine results > 1.2 mg/dL were identified as possible CKD patients by ID-LCMS creatinine, indicating a CKD prevalence of approximately 7.8%. The mean of duplicate readings was use for data analysis of BCMS, and Cobas Integra results. For the Dimension RxL and Vitros DT60 single readings, only singleton plasma readings were undertaken.

### Control results for DBS method

LCMS testing of the two control DBS samples for sample stability testing showed that there was minimal influence of shipment and storage on creatinine measurement values, with a mean percent difference pre- and post-storage of 1.87%. Analysis of the replicate testing of the controls by LCMS to compare whole blood creatinine, plasma creatinine, and DBS creatinine values showed differences in creatinine levels between the three sample preparations. The mean ratio of DBS creatinine values to whole blood creatinine values was 1.16. This difference relates to the variability associated with the DBS sample volume extracted for LCMS testing. The DBS method assumes that a 6-mm DBS is equivalent to a 10-µL whole blood sample. Previously, we have found that a 6-mm DBS is equivalent to 11 12 µL of whole blood which has also been reported in a recent study [36]. Based on this ratio, a correction factor (0.862) was applied to the patient DBS LCMS creatinine results.

### Alignment of methods

**Fig. 1:**
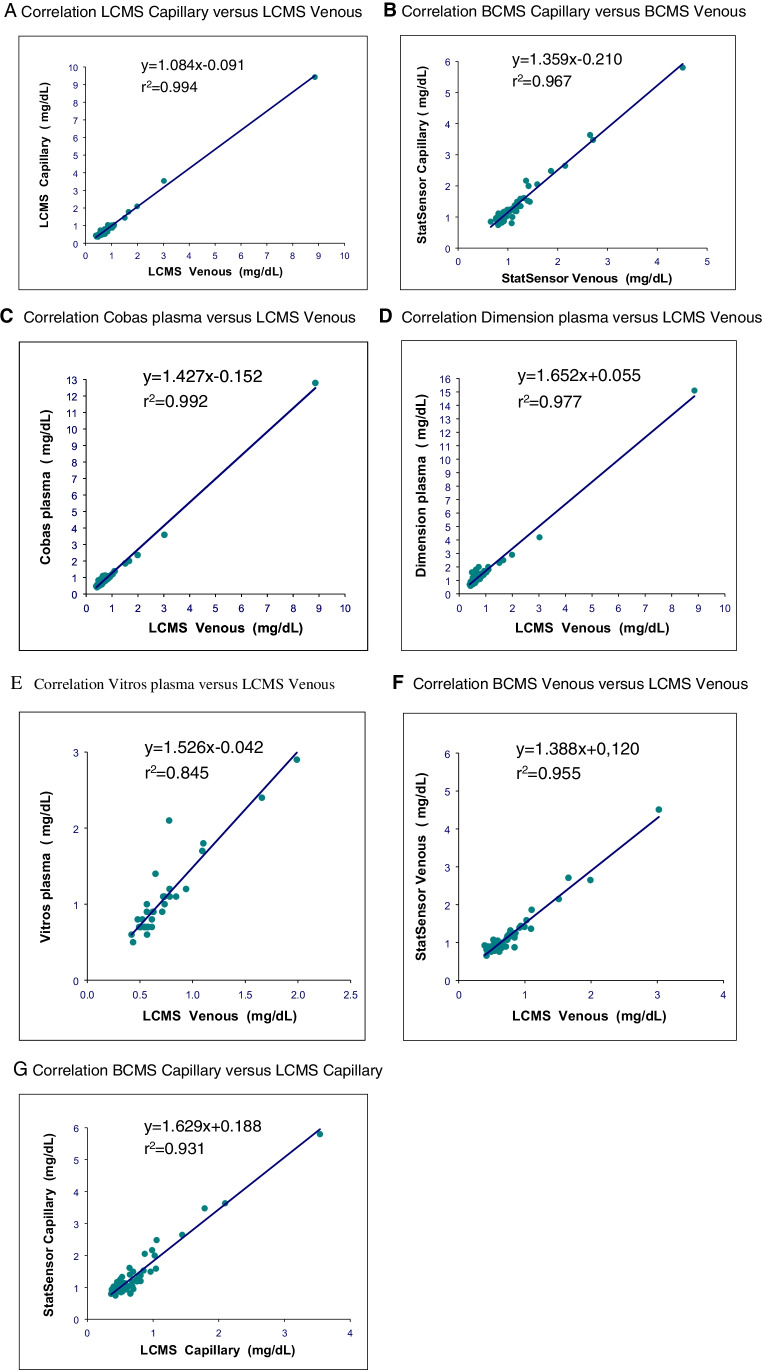
Linear regression analyses for each specimen type and creatinine method compared to ID-LCMS. **A** Correlation LCMS capillary versus LCMS venous. **B** Correlation BCMS capillary versus BCMS venous. **C** Correlation Cobas plasma versus LCMS venous. **D** Correlation dimension plasma versus LCMS venous. **E** Correlation Vitros plasma versus LCMS venous. **F** Correlation BCMS venous versus LCMS venous. **G** Correlation BCMS capillary versus LCMS capillary

**Fig. 2:**
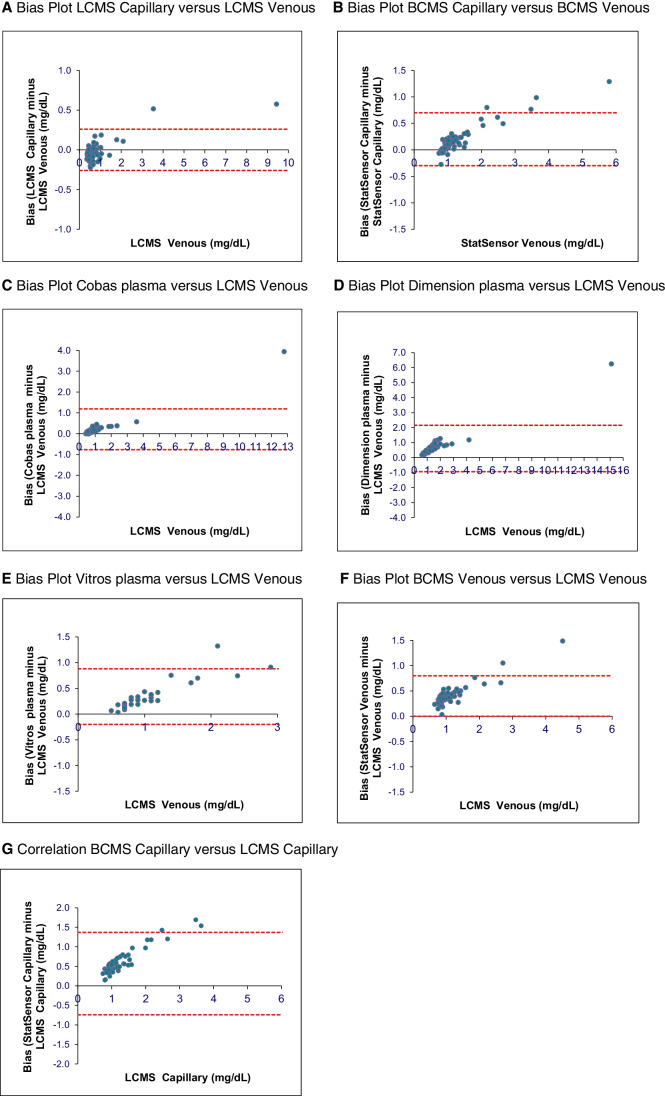
Bias plots of each creatinine method compared to ID-LCMS. **A** Bias plot LCMS capillary versus LCMS venous. **B** Bias plot BCMS capillary versus BCMS venous. **C** Bias plot Cobas plasma versus LCMS venous. **D** Bias plot dimension plasma versus LCMS venous. **E** Bias plot Vitros plasma versus LCMS venous. **F** Bias plot BCMS venous versus LCMS venous. **G** Correlation BCMS capillary versus LCMS capillary

The BCMS’s upper limit of detection is 12 mg/dL. Capillary and corresponding venous whole blood specimen results > 12 mg/dL were recorded as high and were not included in the LCMS method comparison correlation analysis. Only 34 venous creatinine measurements were available for the Vitros analyzer. There was a close correlation between capillary and venous LCMS creatinine results with a mean absolute bias of − 0.02 mg/dL (Table 1 and Figs. 1a and 2a). There was also good correlation between capillary and venous whole blood BCMS results, with a mean absolute bias of 0.19 mg/dL (Table 1 and Figs. 1b and 2b).

**Table 1:** Correlation of creatinine testing methods

	Regression analysis				Bias (method–reference)	
	*n*	*r*^2^	Slope	Intercept	Mean (mg/dL)	SD (mg/dL)
LCMS capillary versus LCMS venous	64	0.994	1.084	− 0.091	− 0.02	0.13
Roche Cobas plasma versus LCMS venous	64	0.992	1.427	− 0.152	0.21	0.49
Siemens RxL plasma versus LCMS venous	64	0.977	1.652	0.055	0.61	0.77
Vitros plasma versus LCMS venous	34	0.845	1.526	− 0.042	0.34	0.27
BCMS venous versus LCMS venous	63	0.955	1.388	0.120	0.40	0.20
BCMS capillary versus LCMS capillary	63	0.931	1.629	0.188	0.63	0.37
BCMS capillary versus SSC venous	64	0.967	1.359	− 0.210	0.19	0.25

Plasma creatinine results from three laboratory analyzer methods correlated closely to the ID-LCMS method, as did both the BCMS capillary and venous whole blood creatinine results (Table 1). Creatinine measurements were higher for the laboratory and POC methods with mean absolute bias differences ranging from 0.21 to 0.63 mg/dL (Fig. 2).

### Sensitivity and specificity analysis

**Fig. 3:**
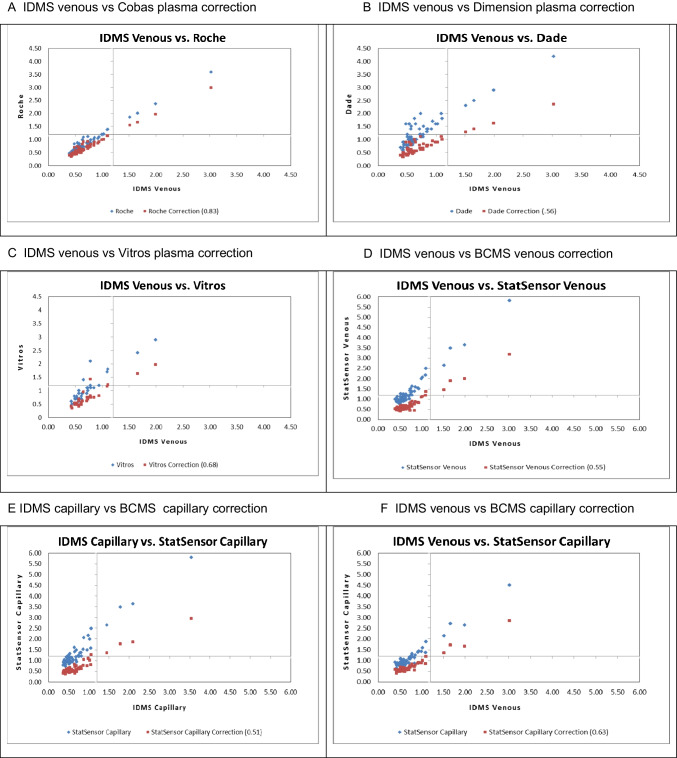
Distribution of creatinine values pre- and post-correction for each specimen type and creatinine method compared to ID-LCMS. **A** IDMS venous vs Cobas plasma correction. **B** IDMS venous vs dimension plasma correction. **C** IDMS venous vs Vitros plasma correction. **D** IDMS venous vs BCMS venous correction. **E** IDMS capillary vs BCMS capillary correction. **F** IDMS venous vs BCMS capillary correction

All methods identified the elevated creatinine results, yielding 100% sensitivity for each method utilized in the study to detect a patient’s CKD risk (Table 2). However, due to the differences in mean percent bias, the specificity of the methods varied from 62.7 to 94.9% with positive predictive values (PPV) ranging from 25 to 62.5%. A correction factor (presented in Fig. 3 for each method) was applied to align all the data from each method to the ID-LCMS method to compensate for the biases in the calibration of each method. The alignment of creatinine values pre- and post-correction for each specimen type and creatinine method compared to ID-LCMS are presented in Fig. 3. This mathematical alignment of the data improved specificity considerably without affecting sensitivity (Table 2). For the BCMS, the specificity increased from 83.1 to 100% for venous whole blood creatinine results and 72.1 to 98.3% for capillary whole blood creatinine results. Mathematical alignment of each plasma creatinine method also improved each method’s specificity: Cobas Integra 400 improved from 94.9 to 100%, Dimension RxL improved from 62.7 to 100%, and Vitros DT60 improved from 81.3 to 96.9%.

**Table 2:** Creatinine method concordance: pre- and post-calibration offset alignment at decision making level of 1.2 mg/dL

Method		Sample	Sensitivity	Specificity	PPV	NPV
*Venous blood*						
Roche Cobas	Pre-offset	64	100 (5/5)	94.9 (56/59)	62.5	100
	Post-offset (0.83)	64	100 (5/5)	100 (59/59)	100	100
Siemens RxL	Pre-offset	64	100 (5/5)	62.7 (37/59)	18.5	100
	Post-offset (0.58)	64	100 (5/5)	100 (59/59)	100	100
Vitros	Pre-offset	35	100 (3/3)	81.3 (26/32)	33.3	100
	Post-offset (0.68)	35	100 (3/3)	96.9 (31/32)	75.0	100
BCMS	Pre-offset	64	100 (5/5)	83.1 (49/59)	33.3	100
	Post-offset (0.63)	64	100 (5/5)	100 (59/59)	100	100
*Capillary blood*						
BCMS	Pre-offset	64	100 (5/5)	72.1 (44/59)	25.0	100
	Post-offset (0.51)	64	100 (5/5)	98.3 (58/59)	83.3	100

## Discussion

Despite an international drive to improve the standardization of creatinine measurement methods [20], an international study involving assessment of external equality assessment samples reported that variability in creatinine measurements is still a concern [21]. More recently, a Canadian study also reported significant inter-laboratory variability indicating that improvements in creatinine assay performance and alignment are needed [34]. Working reference material from NIST is available to achieve IDMS calibration alignment for standardization purposes. However, an assessment of creatinine methodology used for CKD prevalence studies reported that only 29% of studies referred to the use of IDMS calibration [35]. Without confirmed traceability to a higher order reference method, it is difficult to interpret the outcome of CKD prevalence studies. IDMS alignment and whole blood testing commutability is important when using any capillary or venous whole blood specimen for BCMS creatinine to determine CKD prevalence at the point of care. To date, for CKD prevalence field studies, we are not aware of direct comparison studies that compare capillary BCMS results to ID-LCMS results. In this study, both whole blood capillary and venous specimens were collected and processed as air DBS and sent for ID-LCMS testing. Additionally, method and QC process testing was undertaken prior to ID-LCMS patient testing to validate the stability of DBS sample shipment, storage, and testing later to the initial field and laboratory testing in Nicaragua. Although other studies report using DBS to check creatinine, these are not common, and this study validates DBS use for creatinine measurement, making testing in remote or underserved areas feasible [37, 38].

In the initial publication of the field study performed to assess the BCMS for monitoring CKD in Nicaragua [23], the authors reported a sensitivity of 100% and a specificity of 79% compared to the laboratory reference method (Roche Cobas’ compensated Gen 2 Jaffé method) in use. In this follow-up analysis, the laboratory method results, along with the results of two additional laboratory methods, were not mathematically aligned or commutable to the ID-LCMS method results and, as such, demonstrated varying specificity when compared to the ID-LCMS method. Following mathematical re-calibration and alignment of each method to the ID-LCMS method, the specificity and PPV of all methods improved considerably. Consequently, the specificity of the BCMS venous and capillary blood testing post-calibration alignment was 100% and 98.3% respectively, indicating that the device is suitable to screen for CKD in POC settings and is a reliable method to assess a patient’s renal status in the field. The importance of establishing ID-LCMS traceability and alignment prior to undertaking CKD studies is now clearly established. We acknowledge that this study was not sufficiently powered with normal and abnormal patient participation to represent the five CKD stages. Unfortunately, we were unable to obtain patient blood pressure or weight, height, and BMI to calculate eGFR for each patient as an additional metric to assess the BCMS’ concordance to the CKD stages [34]. In this study, comparison of ID-LCMS capillary and venous results showed a lower mean absolute bias (− 0.02 mg/dL) than the comparison of the BCMS capillary to venous results (0.19 mg/dL). The primary reason for these differences is most likely due to the variability caused by capillary skin puncture specimen collection. It is possible that the timing of when the specimens were collected to when they were analyzed played a role in this bias, given the known interference of pyruvate in the Jaffe method [19], and the conversion of pyruvate to lactate over time in whole blood [39]. Despite this increased variability from skin puncture collection, capillary whole blood testing with a sensitivity of 100% is acceptable for CKD screening and a specificity of 98.3% after IDMS alignment confirmed stage 5 CKD in 5 patients. It is important to note that the lab method must first be aligned to IDMS with subsequent alignment of the BCMS method to the lab method to achieve good analytical and clinical agreement.

## Conclusion

We have presented a unique new approach to determine the alignment of a whole blood POC testing creatinine methodology to ID-LCMS. As evidenced by the data from all methods studied, alignment to ID-LCMS will improve the outcome and estimation of CKD in prevalence epidemiological studies, as well as health screening at the point of care to identify patients more readily with CKD at onset. The reliability of studies to assess CKD at the point of care is improved when ID-LCMS alignment is established.

## Abbreviations

BCMS: Blood creatinine monitoring systems
CKD: Chronic kidney disease
DBS: Dried blood spots
ESRD: End-stage renal disease
ID-LCMS: Stable isotope dilution liquid chromatography mass spectrometry
IDMS: Stable isotope dilution mass spectrometry
NPV: Negative predictive value
POC: Point of care
PPV: Positive predictive value
RRT: Renal replacement therapy
RT: Room temperature

## Declarations

This study was conducted under IRB approval and informed consent was obtained from participants prior to sprecimen collection.

Nova Biomedical provided equipment and laboratory technical support for both field and confirmation testing including payment for mass spectrometry testing.
